# Genetics of amyotrophic lateral sclerosis: seeking therapeutic targets in the era of gene therapy

**DOI:** 10.1038/s10038-022-01055-8

**Published:** 2022-06-13

**Authors:** Naoki Suzuki, Ayumi Nishiyama, Hitoshi Warita, Masashi Aoki

**Affiliations:** Department of Neurology, Tohoku University Graduate School of Medicine, 1-1 Seiryo-machi, Aoba-ku, Sendai, Japan

**Keywords:** Motor neuron disease, Medical genetics

## Abstract

Amyotrophic lateral sclerosis (ALS) is an intractable disease that causes respiratory failure leading to mortality. The main locus of ALS is motor neurons. The success of antisense oligonucleotide (ASO) therapy in spinal muscular atrophy (SMA), a motor neuron disease, has triggered a paradigm shift in developing ALS therapies. The causative genes of ALS and disease-modifying genes, including those of sporadic ALS, have been identified one after another. Thus, the freedom of target choice for gene therapy has expanded by ASO strategy, leading to new avenues for therapeutic development. Tofersen for superoxide dismutase 1 (SOD1) was a pioneer in developing ASO for ALS. Improving protocols and devising early interventions for the disease are vital. In this review, we updated the knowledge of causative genes in ALS. We summarized the genetic mutations identified in familial ALS and their clinical features, focusing on *SOD1*, fused in sarcoma *(FUS)*, and transacting response DNA-binding protein. The frequency of the *C9ORF72* mutation is low in Japan, unlike in Europe and the United States, while *SOD1* and *FUS* are more common, indicating that the target mutations for gene therapy vary by ethnicity. A genome-wide association study has revealed disease-modifying genes, which could be the novel target of gene therapy. The current status and prospects of gene therapy development were discussed, including ethical issues. Furthermore, we discussed the potential of axonal pathology as new therapeutic targets of ALS from the perspective of early intervention, including intra-axonal transcription factors, neuromuscular junction disconnection, dysregulated local translation, abnormal protein degradation, mitochondrial pathology, impaired axonal transport, aberrant cytoskeleton, and axon branching. We simultaneously discuss important pathological states of cell bodies: persistent stress granules, disrupted nucleocytoplasmic transport, and cryptic splicing. The development of gene therapy based on the elucidation of disease-modifying genes and early intervention in molecular pathology is expected to become an important therapeutic strategy in ALS.

## Introduction

Amyotrophic lateral sclerosis (ALS) is the most common motor neuron disease (MND) among adults [1, 2]. No treatment, other than symptomatic management for dysphagia and respiratory failure, has been established. The pathomechanism of ALS has been elucidated by functional analysis of genes identified in familial ALS, which occurs in approximately 10% of patients with ALS. As many disease susceptibility genes have been reported in recent studies, genetic factors are now considered significantly involved in sporadic ALS [3].

Spinal muscular atrophy (SMA) is a motor neuron disease caused by a decrease in the survival motor neuron (SMN) protein due to *SMN1* deficiency. Although SMN2, which is almost identical to SMN1, is present in vivo, mRNA from the gene is usually skipped in exon 7 due to splicing, and little functional SMN is synthesized. Nusinersen is a chemically-modified RNA that targets intronic splicing silencer N1 in intron 7 of *SMN2* and inhibits the skipping of exon 7, thereby allowing the synthesis of functional SMN proteins from *SMN2* [4]. In a randomized, double-blind, placebo-controlled trial (ENDEAR trial: NCT02193074) involving patients with infantile-onset SMA (type 1), nusinersen-treated patients showed a significant reduction in mortality and improvement in motor function [5]. Moreover, nusinersen has been shown to be effective in treating type 2 and type 3 SMA (CHERISH trial: NCT02292537) [6].

The approval of antisense oligonucleotides (ASOs) for treating SMA has had a significant impact and has brought hope to the development of therapies for other MNDs. Tofersen for superoxide dismutase 1 (SOD1) was a pioneer in developing ASO therapies for ALS [7]; however, it failed to show significant improvement in the primary endpoint in a phase 3 trial [8]. Therefore, improving protocols and developing early interventions for the disease are vital.

In this review, we have updated the knowledge of causative genes in ALS. Moreover, we have summarized the genetic mutations identified in familial ALS and their clinical features, focusing on *SOD1*, fused in sarcoma *(FUS)*, and transacting response DNA-binding protein *(TARDBP)*. A genome-wide association study (GWAS) has revealed disease-modifying genes that could be the target of gene therapy. The current status and prospects of gene therapy development were discussed, including ethical issues. Furthermore, we have discussed the potential of axonal pathology as a new therapeutic target from the perspective of early intervention.

## The elucidation of causative genes has advanced our understanding of the pathogenesis of ALS, and genetic analysis has become an essential tool for developing personalized treatment

### More than 30 ALS-causing and related genes have been identified

The identification of the *SOD1* gene as the causative gene in 1993 was a major step forward in the study field of ALS [9–11]. Moreover, the *TARDBP* and *FUS* genes have been identified, and RNA metabolism has become a focus of attention as a pathological factor in ALS [12–15]. In 2001, ALS2/Alsin was identified as the causative gene for a young-onset autosomal recessive form of ALS [16]. In 2010, optineurin was identified as a cause of ALS with slow progression, extended duration, lower limb onset with spasticity, and cognitive impairment [17]. Both alsin and optineurin are autophagy-related molecules, suggesting that disruption of the protein degradation machinery is involved in the pathogenesis of ALS [18, 19].

In 2011, chromosome 9 open reading frame 72 (*C9ORF72*), the most frequent causative gene in familial ALS in Europe and the United States, was identified, which markedly changed the research trend [20, 21]. A *C9ORF72* mutation has been described as frontotemporal dementia-ALS 1 (FTDALS1), which is found in approximately 40% of patients with familial ALS and 3% of patients with sporadic ALS in Europe and the United States [22]. Targeted resequencing and exome analysis of familial ALS using next-generation sequencers have been reported one after another and revealed novel causative genes [23–26]. As of December 2021, the Online Mendelian Inheritance in Man (OMIM) has registered 26 types of ALS and 24 causative genes, except for ALS3 and ALS7 (Table 1). ALS13, ALS24, and ALS25 have been suggested to be susceptibility genes (# in Table 1).

**Table 1:** Updated list of ALS causative and modifying genes [24, 25, 327]

ALS type	Gene name	Inheritance	Locus	Onset age	Clinical features	Selected molecular pathology
ALS1	SOD1 (superoxide dismutase 1)	AD	21q22.11	adult	Most frequent in Japanese familial ALS. LMN dominant case. Rare cognitive impairment.	oxidative stress, ER stress, non-autonomous cell death, axon pathology
ALS2	ALSIN (KIA1563)	AR	2q33.1	juvenile	infancy to mid-childhood onset. UMN dominant. Not observed in adult-onset ALS.	vesicle trafficking, autophagy, activation of Rab proteins
ALS3	Unknown	AD	18q21	adult	typical ALS. 1 family in Europe.	Unknown
ALS4	SETX (senataxin)	AD	9q34.13	juvenile	Also referred to as distal hereditary motor neuropathy. No bulbar involvement. AOA2 and SPG19 are allelic disorders.	DNA repair
ALS5	SPG11 (Spatacsin)	AR	15q21.1	juvenile	Very slowly progressive. No cognitive impairment or thin corpus callosum. CMT2X and SPG11 are allelic disorders.	axon development, DNA repair
ALS6	FUS (fused in sarcoma)	AD	16p11.2	juvenile-adult	Second most in Japanese familial ALS. Rapid progression. With or without cognitive impairment. Case with Postural tremor and an autism spectrum disorder.	RNA metabolism, stress granule, DNA repair, axon pathology
ALS7	Unknown	AD/AR	20p13	adult	1 family in the US	Unknown
ALS8	VAPB (vesicle-associated membrane protein B)	AD	20q13.3	adult	7 families were primarily reported in Brazil. LMN is dominant. Postural tremor.	vesicle trafficking
ALS9	Angiogenin	AD	14q11.2	adult	Typical ALS. May present with FTD or parkinsonism.	angiogenesis
ALS10	TARDBP (TAR DNA-binding protein 43)	AD	1p36.22	adult	Third most frequent in Japanese familial ALS. Coding TDP-43. Usually slow progressive. p.G376D is rapid progressive.	RNA metabolism, stress granule, axon pathology,
ALS11	FIG4 (phosphoinositide 5-phosphatase)	AR	6q21	adult	Bulbar onset in half of patients. CMT4J is an allelic disorder.	vesicle trafficking
ALS12	OPTN (Optineurin)	AD/AR	10p13	juvenile	Slow progression and extended duration. Lower limb onset with spasticity. Cognitive impairment.	autophagy, neuroinflammation
ALS13#	ATXN2 (ataxin-2)	AD	12q24.12	adult	Typical ALS. CAG repeat 27-33 is a risk for ALS. > 32 repeats could be SCA2.	TDP-43 proteinopathy, stress granule
ALS14/FTDALS6	VCP (valocin-containing protein)	AD	9p13.3	adult	May rapid clinical course and include parkinsonism. MSP1.	protein degradation
ALS15	UBQLN2 (ubiquilin-2)	XL	Xp11.21	juvenile-adult	Onset varies from childhood to late adulthood. UMN dominant.	protein degradation
ALS16	SIGMAR1 (sigma non-opioid intracellular receptor 1)	AR	9p13.3	juvenile	Slow progression. Lower limb onset with spasticity.	ER stress
ALS17/FTDALS7	CHMP2B (charged multivesicular body protein 2B)	AD	3p11.2	adult	LMN dominant	vesicle trafficking, protein degradation
ALS18	PFN1 (profilin 1)	AD	17p13.2	adult	Limb onset. Feature of Miller-Dieker syndrome (lissencephaly).	cytoskeleton, axon elongation
ALS19	ERBB4 (chorion protein gene ErB.4)	AD	2q34	adult	relatively late onset	tyrosine kinase
ALS20	HNRNPA1 (heterogeneous nuclear ribonucleoprotein A1)	AD	12q13.13	adult	IBMPFD3/MSP3 are allelic disorders. Families restricted to Inclusion body myopathy are reported.	RNA metabolism, nucleocytoplasmic transport
ALS21	MATR3 (matrin-3)	AD	5q31.2	adult	VCPDM (vocal cord and pharyngeal dysfunction with distal myopathy) or MSP5. slowly progressive.	protein degradation
ALS22	TUBA4A (tubulin alpha 4A)	AD	2q35	adult	typical ALS	Cytoskeleton
ALS23	ANXA11 (annexin A11)	AD	10q22.3	adult	typical ALS	vesicle trafficking
ALS24#	NEK1 (never in mitosis gene A-related kinase 1)	AD	4q33	adult	slow progression and extended duration. Spondylometaphyseal dysplasia is an allelic disorder.	DNA repair, cytoskeleton, cilia, cell cycle
ALS25#	KIF5A (kinesin superfamily protein 5A)	AD	12q13.3	adult	young adult. SPG10 is an allelic disorder.	motor protein, axonal pathology
ALS26	TIA1 (T cell intracellular antigen-1)	AD	2p13.1	adult	with or without FTD	Stress granule
FTDALS1	C9ORF72 (chromosome 9 open reading frame 72)	AD	9p21.2	adult	Most frequent in Europe/US. Founder effect. GGGGCC hexanucleotide repeat expansion. Non-AUG translation. Dipeptide repeat protein aggregation.	RNA metabolism, autophagy, immune modulation, neuroinflammation, nucleocytoplasmic transport
FTDALS2	CHCHD10 (coiled-coil-helix-coiled-coil-helix domain containing protein 10)	AD	22q11.23	adult	Parkinsonism, sensory hearing impairment, myopathy, or cerebellar ataxia.	mitochondria
FTDALS3	SQSTM1 (sequestosome-1) / p62	AD	5q35.3	adult	found in FUS-ALS, Alzheimer's dementia, or Paged disease. MSP4.	protein degradation, autophagy
FTDALS4	TBK1 (TANK-binding kinase 1)	AD	12q14.2	adult	mild cognitive impairment. Reduced penetrance.	NFkB, neuroinflammation, autophagy
FTDALS5	CCNF (Cyclin-F)	AD	16p13.3	adult	typical ALS	E3 ligase, cell cycle, autophagy
FTDALS8	CYLD (CYLD Lysine 63 Deubiquitinase)	AD	16q12	adult	FTD	deubiquitination
NA	SPTLC1 (Serine Palmitoyltransferase Long-Chain Base Subunit 1)	de novo	9q22	juvenile	juvenile ALS	sphingolipid synthesis pathway
NA	HNRNPA2B1 (heterogeneous nuclear ribonucleoprotein A2B1)	AD	7p15.2	adult	MSP2	RNA metabolism, nucleocytoplasmic transport
NA	C21orf2 (chromosome 21 open reading frame 2)	ND	21q22.3	adult	typical ALS. Spondylometaphyseal dysplasia is an allelic disorder.	Cilia, Cytoskeleton, NEK1 mediated
NA#	UNC13A	ND	19p13	adult	susceptibility gene	synaptic function
NA	DCTN1 (dinactin-1)	AD/AR	2p13.1	juvenile	slowly progressive, parkinsonism	axonal transport
NA	TFG (TRK-fused gene)	AD/AR	3q12.2	adult	motor and sensory neuropathy	ER stress, axonal pathology

Clinical features and molecular pathogenesis hypothesis of ALS-related genes. This table summarizes the major ones described in relation to ALS in Online Mendelian Inheritance in Man (https://www.omim.org/:OMIM) as of December 2021. Clinical features and molecular functions and associated molecular pathogenesis have been extracted from previous literature. # are interpreted as disease susceptibility genes

*NA* not available, *AD* autosomal dominant, *AR* autosomal recessive, *LMN* lower motor neuron, *UMN* upper motor neuron, *AOA* ataxia with oculomotor apraxia, *SPG* spastic paraplegia, *CMT* Charcot–Marie–Tooth, *MSP* multisystem proteinopathy, *IBMPFD* inclusion body myopathy with Paget’s disease of the bone and frontotemporal dementia, *VCPDM* vocal cord and pharyngeal dysfunction with distal myopathy

### Racial/ethnic differences in genetic analysis

How often are these genetic mutations found in familial and sporadic ALS in Japan? The Japanese Consortium of ALS Research (JaCALS) has found known mutations in 48.7% of 39 Japanese families with suspected familial ALS, mainly in the autosomal dominant form, and known ALS-causing gene mutations in 3% of 469 cases of sporadic ALS [27]. We have been studying familial ALS since 1991. We performed targeted resequencing analysis of 111 Japanese families with suspected autosomal dominant forms of ALS [28]. We identified *SOD1* mutations in 36 families, *FUS* mutations in 12 families, *TARDBP* mutations in two families, and optineurin p.E478G mutations in one family. We identified known mutations in 50% of the families with familial ALS [28].

**Fig. 1:**
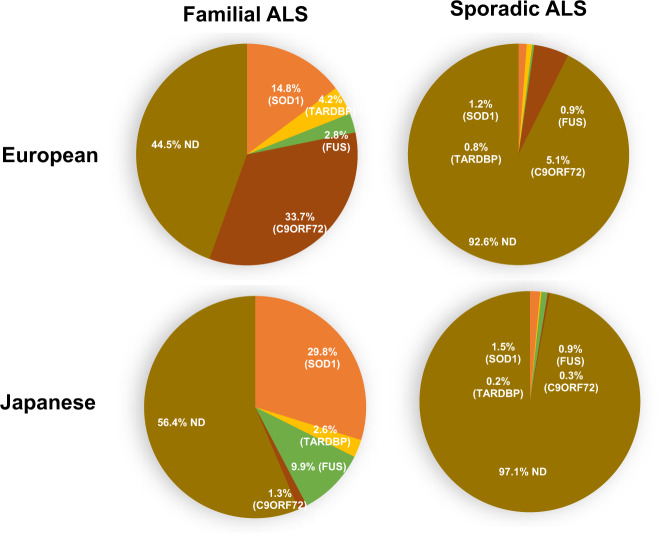
Racial/ethnic difference of amyotrophic lateral sclerosis (ALS) causative genes [28, 29, 93]. The pie charts of ALS causative genes in Europeans and Japan are shown, color-coded with *SOD1, TARDBP, FUS, C9ORF72*, and not determined (ND) in these four genes. Mutations were identified in 55.5% of Europeans with familial ALS and 43.6% of Japanese individuals with familial ALS. In sporadic ALS, only 7.4% of mutations were identified in Europe and 2.9% in Japan. The difference between European and Japanese is largely due to the difference in the frequency of *C9ORF72* mutation, *SOD1*, and *FUS* being more common in Japanese and *TARDBP* being more common in European

The results of the analysis of causative genes in European and Japanese are shown in Fig. 1. Mutations in *SOD1, TARDBP, FUS*, and *C9ORF72* were color-coded according to a review of the literature [27, 29], and data in Japan is modified from our previous study [28]. Mutations not determined (ND) in these four genes were also color-coded. Mutations were identified in 55.5% of Europeans with familial ALS and 43.6% of Japanese individuals with familial ALS. In sporadic ALS, mutations were also identified in 7.4% of European cases and 2.9% of Japanese cases. The difference between European and Japanese cases is mostly due to the difference in the frequency of *C9ORF72* mutation. Furthermore, *SOD1* and *FUS* are more common in Japanese, while *TARDBP* is more common in European. Asia represents over 50% of the world’s population; however, this continent is underrepresented in clinical trials and studies [30]. We want to point out that the frequency of the *C9ORF72* mutation is low in Japan, unlike in Europe and the United States, while *SOD1* and *FUS* are more common, indicating that the target mutations for therapy vary by ethnicity.

### Clinicogenetic and molecular characteristics of ALS genetic variants

#### SOD1

SOD1 (Cu/Zn-SOD) is a protein consisting of 153 amino acids, and more than 200 *SOD1* mutations have been reported worldwide [9, 10]. Many cases are clinically indistinguishable from sporadic ALS, except for family history. The progression of disease with mutations of *SOD1* is well correlated with each mutation [31, 32]. Most cases start in the lower motor neurons and lower limbs [33]. A correlation was found between certain point mutation and symptom severity, with p.A4V indicating severe disease and p.H46R indicating slow progression [34–36]. In our cohort, the mean age at onset was 48.4 years, and the mean disease duration was 4.9 years in patients with *SOD1* mutations. The p.L126S mutation is characterized by rapid progression in homozygous cases [37] and relatively long course in heterozygous cases [38], with isolated inferior olivary hypertrophy in autopsy case [38]. The relatively frequent p.N86S mutation is characterized by phenotypic diversity and low penetrance even within families. p.L8V, a rare mutation that causes sensory disturbances, has also been reported [39]. Three mutations, p.H46R, p.L126S, and p.N86S, account for ~40% of the Japanese cases, with a lesser frequency of the severe form, p.A4V, which is estimated to account for 50% of *SOD1* mutations among Europeans and Americans [40]. The p.D90A mutation is frequent in Europe [41], and is also transmitted in the AR form, but has not been found in our Japanese subjects [42]. These regional differences in *SOD1* mutations should be taken into account in the development of *SOD1*-targeted therapies, such as the high prevalence of severe p.A4V in North America, p.D90A in Europe, and slower p.H46R in Japan [43, 44].

*The current HGVS nomenclature uses one amino acid shift compared to past nomenclature [45]. However, this review follows the traditional numbering for SOD1 variants in order to be consistent with previous articles.

#### FUS

In 2009, *FUS* was identified as the causative gene of ALS [12, 13]. *FUS* is the fourth most common causative gene for familial ALS in the US and Europe, following *C9ORF72/SOD1/TARDBP*. *FUS* mutation frequency is especially high in sporadic, early onset (<35 years of age) ALS patients because of de novo mutations [46]. In the original report, the average age at onset was 44.5 years and average survival was 33 months in familial ALS with *FUS* gene mutations, indicating early onset and rapid progress disease course [13].

In Japan, *FUS* mutations are the second most frequent ALS-causing gene mutations after *SOD1* mutations in familial ALS [28, 47]. We have identified 11 mutations in 15 families, concentrated on Ex14, and 15 of *FUS* (Table 2) [48]. The *FUS* mutations tended to occur at a relatively young age, in the 30 or 40 s, with a cervical or upper extremity onset and a fast progressive disease course of ~2 years [48]. In contrast, families with the p.Q519E and p.S513P mutations had an older age of onset, a slightly slower progression rate, and a lower extremity onset. In a large family with the p.R521L mutation in the *FUS* gene, where we could obtain a detailed clinical profile of over five generations, 23 of 46 patients had ALS, and the penetrance rate was estimated to be as high as 100% [47]. At 35.3 years of age, the patients had muscle weakness, dysarthria, dysphagia, muscle spasticity, and atrophy [47]. The average age at death was 37.2 years, and the average time to need a ventilator was 23 months, indicating a young age of onset and a rapidly progressive course. In three autopsied cases with 1, 3, and 9 years of disease duration, the distribution of FUS-positive cytoplasmic inclusions according to disease stage was widespread [49]. In the case with a 9-year disease duration, in addition to the usual findings of ALS, histological examination revealed atrophy of the midbrain capsules, extensive neuronal loss of the substantia nigra, nucleus accumbens, subthalamic nucleus, and globus pallidus, especially the medial segment [49]. Cases with postural tremor and autism spectrum disorder were also reported [50].

**Table 2:** FUS mutations and their clinical features in our cohort [28, 48]

No.	Exon	Mutation		Age at onset	Duration (month)	Site of onset	Cognitive decline
1	14	p.G472VfsX57	c.1420_1421 ins GT	26	12	L/E	-
2	14	p.G497AfsX527	c.1485 del A	18	11	Neck, U/E(P)	MR
3	14	p.K510E	c.1528 A > G	28	10	Neck	-
4	14	p.S513P	c.1537 T > C	62	84	L/E	-
5	14	p.S513P	c.1537 T > C	52	156	L/E	-
6	14	p.R514S	c.1542 G > C	42	43	Bulbar	-
7	14	p.R514S	c.1542 G > C	51		Bulbar, U/E(P)	-
8	15	p.H517P	c.1550 A > C	31	24	Bulbar	-
9*	15	p.H517D	c.1551 C > G	39 (*n* = 2)	20	Neck, U/E(P)	-
10	15	p.Q519E	c.1555 C > G	60 (*n* = 2)	alive > 192 ‡	L/E	-
11*	15	p.R521S	c.1561 C > A	35.5 (*n* = 2)	49	Bulbar, U/E(P)	-
12*	15	p.R521L	c.1562 G > T	38.5 (*n* = 2)	17	Neck, U/E(P)	FTD
13*	15	p.R521L	c.1562 G > T	35.7 (*n* = 13)	17.7	Neck, U/E(P)	-
14	15	p.R521L	c.1562 G > T	44		Neck	-
15	15	p.R521H	c.1562 G > A	39		L/E	-
			Average	40.1	40.3		

We have identified 11 mutations in all 15 families, concentrated in Ex14 and Ex15. Most are young-onset and rapidly progressing, with spherical symptoms and cervical spinal cord region onset. p.Q519E and p.S513P families have older age, slightly slower progression, and lower extremity onset.

*n* number of patients, *FTD* frontotemporal dementia, *L/E* lower extremity, *MR* mental retardation, *U/E(p)* upper extremity with proximal dominancy, − absent

#### TARDBP

In 2008, *TARDBP* (coding TDP-43) was identified as a causative gene of ALS [14, 15]. Before this finding, Arai et al. reported a pioneering biochemical and immunohistochemical analysis of TDP-43 inclusion bodies in autopsied brains of patients with ALS and FTD in 2006 [51]. In Japanese patients with ALS, *TARDBP* is the third most common familial ALS gene after *SOD1* and *FUS* [28]. TDP-43 aggregation in the cytoplasm of spinal anterior horn neurons is a characteristic pathological finding in ALS and is observed in over 90% of patients with sporadic ALS and familial ALS with the *C9ORF72* mutation. Considering the frequency of *TARDBP* mutations in familial ALS and the common TDP-43 pathology between sporadic ALS and familial ALS, focusing on TDP-43 in the pathogenesis of ALS is important. There are few reports of large families [15], and the penetration rate is considered low [52, 53].

Familial ALS with *TARDBP* mutations is more common in the limb onset and has a wider range of onset age [54]. Among these mutations, the p.G376D mutation had a particularly rapid progression, from onset to death in less than 1.5 years [52]. p.G298S mutation is also considered to be short-lived [55], whereas the p.A315T mutation had a longer disease course of 8–10 years. Focusing on rapidly progressing mutations is useful in pathological analysis using cellular and animal models, therefore clinical genetic analysis is important for understanding the pathogenesis of ALS.

#### C9ORF72

The frequency of the *C9ORF72* mutation is high in Europe and the United States, indicating a founder effect of north European (Finnish) origin [56]. It forms a spectrum with FTD, as encoded by FTDALS1 (Table 1). The ALS-causing mutations in *C9ORF72* are hexanucleotide repeat expansion in intron 1 [20, 21]. Bulbar onset has been more frequently observed in *C9ORF72*-mutated ALS [57]. It is still unclear whether anticipation also exists in C9orf72-associated diseases. Somatic and intergenerational repeat instabilities have been observed [57]. The disease penetrance of C9orf72-related ALS is thought to be nearly 100% by the age of 80 [58].

While the frequency of the *C9ORF72* mutation is estimated to be 40% in Europe and the United States, it is less common in Japan, around 1–2% in familial ALS and 0.2% in sporadic ALS [59, 60]. The 20 SNPs in the North European consensus risk haplotype suggest a common ancestry. In the southern part of the Kii Peninsula, an area of high ALS prevalence in Japan, 3 of 15 cases were found to have *C9ORF72* mutation [61]. In our own case, the patient had a typical ALS phenotype with onset between 50 and 70 years of age, frontotemporal dementia, and distal sensory deficits in the lower limbs.

Pathological hypotheses have been proposed: A. loss-of-function of C9ORF72; B. repeat-associated non-AUG (RAN) translation, which is a G4C2 repeat synthesized without the need for a transcription start site; and C. toxicity caused by a dipeptide repeat protein, which is synthesized by the translation of the G4C2 repeat [62]. The C9ORF72 protein is present in motor neurons [63]. C9ORF72 contributes to the maintenance of the immune environment, and its knockout is thought to cause abnormal immune responses, including the release of cytokines, associated with neurodegeneration [64]. Moreover, C9ORF72 knockout mice exhibited strikingly different survival rates depending on their environment and microbiome [65]. G4C2 repeats were transcribed as RNA and accumulated in the nucleus of nerve cells to form RNA foci by a liquid–liquid phase separation (LLPS) mechanism [66]. Furthermore, G4C2 repeat RNA and its translation product, dipeptide repeat protein, have been reported to cause neurodegeneration by increasing DNA double-strand breaks, leading to a deficiency of ataxia telangiectasia mutated (ATM), which repairs DNA damage [67]. Particularly, PR poly-dipeptides are highly toxic because they disrupt nucleocytoplasmic transport by polymerizing with the Nup54 protein in the nuclear pore [68]. A yeast study has found abnormalities in nucleocytoplasmic transport mediated by dipeptide repeat protein toxicity [69]. Furthermore, in a fly model overexpressing a hexanucleotide repeat, the phenotype caused by the *C9ORF72* mutation was alleviated by increasing the expression of RAN GTPase-activating protein 1 (RanGAP1), a key regulator of nuclear and cytoplasmic transport [70]. The deletion of serine/arginine-rich splicing factor 1, which functions as an adapter during the transport of transcribed RNA out of the nucleus, has been reported to suppress neural degeneration by modulating RAN translation and could be a target for novel therapeutic agents [71]. Moreover, ASO, which inhibits repeat RNA, may be used as a therapeutic agent for ALS the *C9ORF72* mutation, and GP repeat dipeptide in cerebrospinal fluid may be an alternative biomarker for determining drug efficacy [72].

#### Multisystem proteinopathy (MSP)

A group of patients known as inclusion body myopathy with Paget’s disease and FTD (IBMPFD) has been described [73, 74]. MSP, which is associated with FTD, inclusion body myopathy, and Paget’s disease of the bone in addition to ALS, has been recognized as an analogous disease concept [75, 76]. Recently, MSPs have been described in the OMIM as MSP1 with valosin-containing protein *(VCP)* mutation, MSP2 with the heterogeneous nuclear ribonucleoprotein *(hnRNP)* A2/B1 mutation, MSP3 with the *hnRNPA1* mutation, and MSP4 with the sequestosome-1 *(SQSTM1)/p62* mutation [77]. The disease concept has been expanded to include *matrin-3* mutations such as MSP5. Theoretically, any of the nearly 50 RNA-binding proteins with prion-like domains, such as FUS, could be a cause of MSPs [78]. *hnRNPA1* mutations may cause only inclusion body myopathy in some families [79, 80]. Furthermore, genetic mutations associated with sensory impairment, parkinsonism, deafness, and various other allelic disorders are associated with MSPs (Table 1). MSP is an important disease concept in the context of motor neuron vulnerability and cell-specific pathology in ALS.

### What GWAS has revealed

GWASs were conducted using single-nucleotide variant arrays to identify disease-associated variations in large cohorts of ALS cases and healthy controls. GWASs can reveal common genetic variants in thousands of unrelated individuals to identify associations with diseases that potentially explain certain percentages of disease heritability within a population [81]. The first GWAS in the ALS field was conducted in 2007, which highlighted the *FLJ10986* gene as a candidate [82]. Following studies have revealed inositol 1,4,5-trisphosphate receptor type 2 [83], dipeptidyl peptidase like 6 [84], unc-13 homolog A (*UNC13A)* [85], Mps one binder kinase activator-like 2B [85], kinesin-associated protein 3 [86], cytochrome P450 family 27 subfamily A member 1 [87], zinc finger protein 512B [88], calcium/calmodulin-dependent protein kinase 1G [89], and sterile alpha and TIR motif-containing protein 1 [90] as representative ALS candidate genes. In 2015, the TANK-binding kinase 1 *(TBK1)* gene was identified as the causative gene for FTDALS4 in an analysis of 2,869 mainly sporadic ALS cases from groups centered in North America [91]. *TBK1* gene variants are found in 1.26% of sporadic ALS in Japan including missense and loss-of-function mutations [92].

A GWAS using 1173 sporadic ALS cases and 8925 controls in a Japanese population combined with a meta-analysis of individuals of European ancestry has revealed a significant association at the Acyl-CoA synthetase long-chain family member 5 *(ACSL5)* locus [93]. A replication study involving a Chinese population and another set of the Japanese populations has confirmed the association. ACSL5 is involved in fatty acid metabolism, and other groups have found an association between *ACSL5* single-nucleotide polymorphisms (SNPs) and lower fat-free mass in patients with ALS [94]. *Serine palmitoyl transferase long-chain base subunit 1* is involved in the sphingolipid synthesis pathway and associated with juvenile ALS [95].

A recent cross-ancestry GWAS involving 29,612 patients with ALS and 122,656 controls identified 15 risk loci [96]. When combined with 6538 patients with whole-genome sequencing and a large cortex-derived expression quantitative trait locus dataset (MetaBrain), analyses have revealed locus-specific rare variants, short tandem repeats, and regulatory effects [96]. The combination of all ALS-associated signals reveals that perturbations contribute to vesicle-mediated transport and autophagy and provides evidence for cell-autonomous disease initiation in glutamatergic neurons [96]. Mendelian randomization analyses, which consider the environmental and lifestyle risk factors obtained from the literature, have indicated that high cholesterol levels play a causal role, again suggesting the importance of lipid metabolism [97]. In another recent analysis, machine learning RefMap method identified risk genes by integrating GWASs and epigenetic data [98]. Convergent genetic and experimental data revealed KN Motif And Ankyrin Repeat Domains 1 *(KANK1)* as a new ALS gene and initiation of ALS pathogenesis in the distal axon [98].

*UNC13A* is a gene repeatedly confirmed in several GWASs [99–104]. The C allele of the rs12608932 SNP within the *UNC13A* gene has been identified as a risk locus for both ALS and FTD [105]. Moreover, this SNP is associated with lower respiratory function at diagnosis and shorter survival [105]. Interestingly, a recent study has revealed that TDP-43 represses a cryptic exon-splicing event in UNC13A and reduces the expression of UNC13A [106, 107]. UNC13A contributes to vesicle priming and controls neurotransmitter release and short-term presynaptic plasticity [108]. UNC13A can be a stratification biomarker and a target of gene therapy. Although it should be noted that odds ratios are usually not high, many genes related to ALS have been identified using GWAS, and progress has been made in understanding the molecular pathogenesis of the disease (Table 3).

**Table 3:** GWASs in the ALS field

Cohort	Size of discovery cohort	Representative identified gene	Role of gene product	References
European	12,577 sporadic ALS (RefMap)	KANK1 (KN Motif And Ankyrin Repeat Domains 1)	cytoskeleton/ actin polymerization	Zhang S (2022)
Cross-ancestry	29,612 ALS	neuron-specific 15 risk loci including UNC13A		van Rheenen W (2021)
European and Chinese	84,694 (meta-analysis)	ACSL5-ZDHHC6	fatty acid metabolism	Iacoangeli A (2020)
Japanese	1173 sporadic ALS	ACSL5 (Acyl-CoA Synthetase Long-Chain Family Member 5)	fatty acid metabolism	Nakamura R (2020)
Caucasian and US	20,806 ALS	KIF5A (Kinesin Family Member 5A)	axonal transport	Nicolas A (2018)
European and Chinese	13,811 sporadic ALS	GPX3(glutathione peroxidase 3)-TNIP1 (TNFAIP3 Interacting Protein 1)	antioxidant and inflammation	Benyamin B (2017)
Australia, European, Turkey	1861 ALS	C21orf2/CFAP410 (Cilia And Flagella Associated Protein 410)	cilia	van Rheenen W (2016)
Caucasian	2869 ALS	TBK1(TANK-binding kinase 1); NEK1 (NIMA Related Kinase 1)	autophagy; cilia/cell cycle	Cirulli ET (2015)
European, US	363 familial ALS probands	TUBA4A (Tubulin Alpha 4a)	cytoskeleton	Smith BN (2014)
European, US	6100 sporadic ALS	SARM1 (Sterile Alpha And TIR Motif Containing 1)	axonal regeneration	Fogh I (2014)
Han Chinese	508 sporadic ALS	CAMK1G	Calcium kinase signaling	Deng M (2013)
Japan	92 ALS	ZNF512B (Zinc Finger Protein 512B)	unknown	Iida A (2011)
European, US	2261 sporadic ALS	CYP27A1 (Cytochrome P450 Family 27 Subfamily A Member 1)	cholesterol metabolism	Diekstra FP (2012)
European, US	1821 sporadic ALS	KIFAP3 (Kinesin-Associated Protein 3)	neurite outgrowth	Landers JE (2009)
European, US	2323 sporadic ALS	UNC13A (Unc-13 Homolog A), MOBKL2B (Mps One Binder Kinase Activator-Like 2B)	synapse formation, kinase activity	van Es MA (2009)
European, US	1767 ALS	DPP6 (Dipeptidyl Peptidase Like 6)	neuronal excitability	van Es MA (2008)
Netherlands	461 ALS	ITPR2 (Inositol 1,4,5-Trisphosphate Receptor Type 2)	Glutamate mediated neurotransmission, apoptosis	van Es MA (2007)
Caucasian	386 sporadic ALS	FLJ10986	carbohydrate phosphorylation	Dunckley T (2007)

GWAS analyses of ALS are presented [81]. Recent studies have included meta-analyses of tens of thousands of patients [94] and weighted analyses focusing on genes expressed in the nervous system [104]. The representative associated genes identified in each study and their functions are listed

### Oligogenic pathogenesis hypothesis

Up to this point, we have assumed a single gene mutation for a single patient. However, although *SOD1* mutations are usually inherited in an autosomal dominant form with high penetrance, there are asymptomatic carriers of some types of mutations [28]. These observation has led to the idea that multiple causative genes cause the disease stage (oligogenic pathogenesis hypothesis), in which gene variants other than *SOD1* are necessary for the onset of the disease [25]. More comprehensive panels of genetic testing will increase the possibility of detecting more than one rare variant in patients with ALS.

The identification of the *C9ORF72* mutation in 2011 furthered this idea of oligogenic pathogenesis. For example, hexanucleotide repeat elongation of *C9ORF72* with variants in other ALS causative genes are associated with a younger age of onset, suggesting that both mutations affect the onset of the disease [109]. Moreover, it can be viewed as a disease susceptibility gene, in the sense that both genetic variants affect the pathogenesis of the disease [28]. Whole-genome sequence of 4,315 cases revealed ALS-associated structural variants including inversion in the *VCP* gene and insertion in the *ERBB4* gene [110]. Over 70% of respiratory onset ALS have *ERBB4* insertion compared with 25% of the control [110]. Answer ALS project revealed 601 expanded regions in the 830 whole-genome sequence data using Expansion Hunter [111]. Large scale whole-genome open resources are now available.

There are other examples where multiple causative genes are associated with faster onset and progression of the disease [112–114]. Mutations in *ataxin-2* cause a polyglutamine chain elongation of 34 repeats or more, which is a phenotype of spinocerebellar ataxia type 2. Ataxin-2 localizes to stress granules, and moderate repeat elongation promotes the activation of caspase 3, which produces TDP-43 C-terminus fragments, leading to ALS [115, 116]. Furthermore, there are ethnic differences in the intermediate-length CAG repeats of *ataxin-2*, with the “large normal allele” being less common in Japanese and more common in non-Japanese populations [117]. *Ataxin-2* poly-CAG expansion is considered the target of ASO [118].

### De novo mutations in sporadic ALS

The reported incidence of some ALS-associated variants in familial and sporadic ALS is different among causative genes. Moreover, 3–16% of sporadic ALS cases have monogenetic etiology (Fig. 1) [28, 29, 93, 119]. Others have reported that 21% of patients with ALS carried a confirmed pathogenic or likely pathogenic mutation, of whom 93% had no family history of ALS [120]. There could be several other cases of sporadic ALS with *SOD1* mutations; however, either (a) DNA analysis of the parents showed one of them to be an asymptomatic mutation carrier; (b) the parents were not the biological parents, or (c) DNA was unavailable from one or both parents. A systematic review and meta-analysis has revealed that the estimated number of patients with *SOD1* or *C9ORF72* mutations are almost the same in familial ALS and sporadic ALS [121], suggesting that familial ALS classification based on reported family history does not capture the full picture of ALS of genetic origin [121].

Family history can be ambiguous or absent because of the following reasons: inadequate family history information in medical charts, misdiagnosis of ALS in older generations, reluctance to report hereditary disease, loss of contact between family members, low penetrance, small family size, early death due to other causes, development of ALS in offspring before the parent who transmitted the defective gene exhibits symptoms themselves, genetic pleiotropy, and lack of information on biological parents (i.e., adoption and illegitimacy) [122]. Moreover, 11.9% of patients carry a clinically relevant genetic mutation, and almost half of the reported mutations in the cohort has a prognostic value [123]. *De novo* mutations in *FUS* were reported in p.R495X and p.P525L cases [124, 125]; these mutations are also found in familial ALS cases. Moreover, de novo mutations in ataxin-2 [126], Erb-B2 receptor tyrosine kinase 4 [127], and Rap guanine nucleotide exchange factor 2 *(RAPGEF2)* have been reported in rare sporadic ALS cases [128]. Possible processes during embryogenesis of a de novo mutation in ALS could be the zygote, epiblast, and ectoderm. If the mutation happens in the ectoderm, we could not detect the mutation in the blood cells, confusing the interpretations.

The finding of de novo mutations in ALS provokes ethical implications. Patients with sporadic ALS who were genetically diagnosed vary in age (e.g., under 40 years old), in whether they have children, and by country (roughly one-third of the patients in Europe and the United States receiving genetic diagnosis) [129]. From the aspect of genetic counseling, it’s important to consider the facts that no fundamental treatment or prevention of the onset of the disease has been established, *de novo* mutations may be passed on to children or grandchildren, and that these facts might cause a large psychological burden. Providing sufficient explanation of the expected benefits and disadvantages before specimen collection and providing sufficient ethical consideration not only to the founder but also to their relatives are necessary. Moreover, collaborating with clinical geneticists and certified genetic counselors in explaining the results is important.

## What is the state of the art in developing therapies targeting genetic mutations?

### Advantages of ASO

The strategy of nusinersen was to compensate for the lack of SMN in SMA. Gene therapy includes introducing functional copies of dysfunctional genes, trophic factors, and disease-modifying genes or silencing the expression of harmful genes. Compared with other drug modalities (e.g., small-molecule or antibody drugs), nucleic acid medicine including ASO is a general term for drugs based on nucleic acids or artificial nucleic acids. Small-molecule drugs have the disadvantage that their drug targets are limited to receptors and enzymes. Antibody drugs are highly evaluated for their specificity and efficacy; however, their drug targets remain limited to molecules expressed on the cell membrane or secreted outside the cell, and mass production is difficult. Nucleic acid drugs have the advantages of being able to target intracellular target molecules, such as mRNA and non-coding RNA, which are difficult to target using conventional drugs, with high specificity and easy to manufacture.

The two main types of siRNAs have been invented: double-stranded RNAs, such as paticiran [130] approved for transthyretin amyloidosis, and single-stranded nucleic acids, such as ASOs. siRNAs currently under development for ALS are ASOs (Table 4). To target the central nervous system, ASOs are delivered either naked or by viral vectors, such as an adeno-associated virus (AAV) [131].

**Table 4:** Clinical trials of gene therapy for ALS

Modality	Target	Trial name	Drug	Mechanism	Phase	Route	References
Antisense oligonucleotides							
	SOD1	VALOR	tofersen (BIIB067)	non-allele specific gapmer (RNase H)	phase III	intrathecal	NCT02623699
	SOD1 (asymptomatic)	ATLAS	tofersen (BIIB067)	non-allele specific gapmer (RNase H)	phase III	intrathecal	NCT0456982
	C9ORF72	245AS101	IONIS-C9 (BIIB078)	expanded repeat specific gapmer (RNase H)	phase I	intrathecal	NCT03626012
	C9ORF72	FOCUS-C9	WVE-004	streopure expanded repeat specific gapmer (RNase H)	phase I/II	intrathecal	NCT04931862
	C9ORF72	TBD	ASO5-2	modification of a subset of the phosphodiester internucleoside linkages	NA	intrathecal	Tran H (2021)
	Ataxin-2	275AS101	ION541 (BIIB105)	non-allele specific gapmer (RNase H) targeting CAG expansion	phase I/II	intrathecal	NCT04494256
	FUS	ION363-CS1	Jacifusen (ION363)	non-allele specific spike-switch ASO (RNase H)	phase III	intrathecal	NCT04768972, Korobeynikov V (2022)
	Stathmin-2	TBD	QRL-201	TBD	phase I	intrathecal	TBD
AAV-mediated delivery							
	SOD1	TBD	APB-102	recombinant AAVrh10 vector that expresses an anti-SOD1 artificial microRNA	Phase I	intrathecal	TBD

Two modalities, ASO and AAV-mediated therapy, have been used in clinical trials. Although *SOD1* and C9ORF72, which have the largest number of ALS cases, have been targeted first, ataxin-2 and FUS are being developed through *n*-of-1 trials. Moreover, targeting stathmin-2, a disease-modifying factor, and UNC13A, a disease-modifying gene (not listed in the table as no information has been published) is planned

### Current status of the therapeutic development of ASOs

Clinical trials of ASO are underway, focusing on SOD1 and C9ORF72, involving several patients. Gene therapy for *SOD1* mutations has been attempted previously [132, 133]. Animal studies have been conducted to suppress the expression of mutant *SOD1* by shRNA transduction using AAV vectors or by genome editing, both of which resulted in phenotypic improvement [132, 133]. Tofersen is an ASO drug being evaluated for the potential treatment of ALS with *SOD1* mutations [7]. VALOR was a phase III, randomized, double-blind, placebo-controlled study that has evaluated the efficacy, safety, tolerability, and pharmacodynamic effects of tofersen on ALS with a confirmed *SOD1* mutation (NCT02623699). Biogen and Ionis have reported that tofersen failed a first phase III trial to prove its effectiveness as the primary endpoint [8]. Early intervention with tofersen might be effective. Asymptomatic patients with known *SOD1* mutations are eligible for the phase III ATLAS study, which examines the pre-symptomatic effect of tofersen, started in May 2021 (NCT0456982).

ASO treatment targeting *C9ORF72*, the most frequent target in Europe and the United States, has been investigated [134]. 245 AS101 is a phase I trial of BIIB078, which targets toxic RNA from hexanucleotide repeats while preserving normal C9ORF72 proteins, administered intrathecally to adults with *C9ORF72*-mutated ALS, sponsored by Biogen (NCT03626012). FOCUS-C9 is a phase Ib/IIa trial of the intrathecal administration of ASO (WVE-004-001) that promotes RNase H-mediated degradation of C9ORF72’s pathogenic mRNA variants associated with ALS or FTD and spares the normal *C9ORF72* V2 variant in neurons (NCT04931862). Recently, the modification of a subset of a phosphodiester internucleotide linker is reported to improve ASO tolerability without impairing potency in repeated dosing for patients with *C9ORF72* mutations [135], though additional clinical trials will be required to prove its efficacy.

The development of therapies for ASO that targets genes other than *SOD1* and *C9ORF72* is also progressing. ION363-CS1 is a phase I–III study that evaluates the efficacy and safety of intrathecally administering ION363/Jacifusen in ALS with *FUS* mutations. First-in-human treatment with ION363 silences FUS expression, decreases FUS pathology, and reverses insolubility of RNA-binding proteins in *FUS*-p.P525L mutated patients [136]. It was started in an *n*-of-1 trial but increased into an international 12-patient study at NEALS active sites (NCT04768972).

Since TDP-43 has been shown to be an important component of ubiquitin-positive and tau-negative inclusion bodies in most ALS cases, including sporadic ALS, the elucidation of the mechanism of abnormal TDP-43 aggregation has become a major research topic. TDP-43 is ubiquitously expressed and plays an important role in RNA metabolism and other cellular functions. Therefore, indirect methods for suppressing TDP-43 toxicity have been explored. For example, the knockdown of TDP-43 fails to maintain the number of motor neurons, and stathmin-2 (STMN2) becomes a mediator [137]. Selected ASOs have high tolerability in rodents but were not tested in monkeys; moderate potency in human motor neurons has been reported. An *n*-of-1 trial of STMN2 ASO was initiated. Initial doses were well tolerated. Although this is an ASO under research, it is the first human tolerability data on STMN2 (Symposium of ALSMND 2021 Dec).

The inhibition of ataxin-2 reduces the abnormal accumulation of TDP-43, prolongs survival, and improves motor function in mice overexpressing mutant *TARDBP* [118]. Moreover, ataxin-2 as an alternative target has attracted much attention [118]. 275AS101 is a phase I trial of BIIB105 targeting poly-CAG expansion in the *ataxin-2* gene to reduce the ataxin-2 protein and mitigate TDP-43 toxicity [118] (NCT04494256).

ASO with constrained ethyl-group chemistry are partially absorbed from the gut [138]. Oral delivery can avoid lumbar-puncture-related adverse events like headaches. Conjugating cholesterol molecules to the ASO might also enhance penetration into the brain and spinal cord after systemic administration [139]. Oral ASO might be the feasible option.

### Therapeutic development using AAV vectors

AAV-based therapies are under active development for various neuromuscular diseases [140]. AAV can be engineered for selective cell targeting and optimized transduction. Without the original viral genome, AAVs are nonpathogenic and unable to replicate like the wild-type virus. AAV9 penetrates the blood–brain barrier (BBB) and targets motor neurons to overcome an obstacle of gene therapy and reveals transduction with tropism with motor neurons [141]. In the case of SMA, onasemnogene abeparvovec (AVXS-101) could deliver across the BBB and into the spinal cord without integrating into the genome of the patient [142] and was approved worldwide. APB-102 is a recombinant AAVrh10 vector that expresses an anti-*SOD1* artificial microRNA (Symposium of ALSMND 2021 Dec) (Table 4).

### Other modalities include antibody drugs and small/medium molecule drugs

Drug discovery in modalities other than ASO is also becoming more active. Antibody drugs are being developed to target various causative gene products. AP-101 is a human monoclonal antibody targeting misfolded SOD1 generated by AL-S Pharma [143] and is now moving toward a human clinical trial. Misfolding-specific intrabody with dual proteolytic signals can eliminate TDP-43 inclusion [144]. Small molecules attached to ribonuclease reduced hexanucleotide repeat expansion in C9ORF72 mouse models [145]. Middle-sized peptides between small molecules and antibodies can be used for targeting “undruggable” intracellular protein–protein interactions [146, 147].

Studies have established the cellular phenotype of motor neurons using induced pluripotent stem cells (iPSCs) derived from patients with familial ALS [148, 149]. Screening of small molecules that improve the survival of motor neurons has also been reported, showing the efficacy of ropinirole and bosutinib, under the concept of drug repositioning [150–152]. A phase II trial of the potassium channel activator retigabine has indicated that short-interval intracortical inhibition as the primary outcome was significantly improved [153]. This work has shown how neurophysiological outcome measures could be used as disease markers.

Let us describe one more trial about developing drug for ALS. The establishment of the mutant *SOD1* transgenic mouse model has greatly advanced research on the pathogenesis of ALS, but the approach to the brainstem and spinal cord, which are the main loci of the disease, has been limited by the small size of the mouse. To overcome the issue of sizing, we created a rat model of mutant *SOD1* transgenes (ALS rats) [154]. The hepatocyte growth factor (HGF) is a novel growth factor originally cloned in Japan [155]. Overexpression of HGF in the nervous system attenuates disease progression and prolongs life span in a transgenic mouse model of ALS [156]. As for the idea of supplementing insufficient factors, intrathecal administration of human recombinant HGF (hrHGF) protein improved motor neuron pathology in a rat model with *SOD1* mutation [154]. Phase II clinical trials are currently underway (UMIN000022050) [157].

The importance of rapid diagnosis and proper evaluation has been recognized in developing any therapeutic approach. Furthermore, the development of appropriate biomarkers and mechanisms to evaluate multiple therapeutic candidates is becoming increasingly important.

### Innovations in biomarkers, clinical trial design, and definition of endpoints

Biomarkers are urgently needed for accurate stratification and diagnosis of patients with ALS for facilitating clinical trials. Moreover, neurofilament light (NFL) and phosphorylated neurofilament heavy chain (pNFH) are biomarkers for ALS [158, 159]. Plasma NFL levels are associated with a higher ALS risk in patients with pre-diagnostic ALS [160]. Plasma pNFH subunit levels are used as a secondary outcome of the trial of sodium phenylbutyrate and taurursodiol [161] or tofersen [7]. miR-181 was reported to have a prognostic value similar to that of NFL [162].

Using spinal cord samples from patients with sporadic ALS and ALS mouse models, vascular cell genes preceded the microglial response even at the pre-symptomatic stage [163]. Secreted phosphoprotein 1 (SPP1)- and COL6A1-positive perivascular fibroblasts accumulated in enlarged perivascular spaces in the spinal cord of patients with sporadic ALS. Increased levels of serum SPP1 could be a biomarker of shorter survival [163]. The combination of NFL and SPP1 or other markers, such as *UNC13A* genotype, might help stratify patients more effectively.

Combined endpoints have been used in several clinical trials to decrease the confounding effect of mortality on the analysis of functional outcomes, though survival and function are assessed as independent endpoints in ALS. The Combined Assessment of Function and Survival (CAFS) ranks patients’ clinical outcomes based on survival time and changes in the ALS Functional Rating Scale-Revised (ALSFRS-R) score [164]. Each patient’s outcome is compared with every other patient’s outcome, a score is assigned, and the summed scores are ranked. A higher mean CAFS score indicates a better group outcome. The CAFS endpoint was used as the primary endpoint of a dexpramipexole phase III study [165] and recent studies [166, 167].

Current clinical trial endpoints may not reflect what patients consider the most important and might estimate the benefit of novel treatments in the wrong way. A new composite endpoint for randomized controlled clinical trials of ALS named the Patient-Ranked Order of Function (PROOF), based on patient preference for functional domains is proposed [168].

In a recent systematic review, among 125 trials, investigating 76 drugs and recruiting more than 15,000 individuals with ALS, ~90% of trials have used traditional fixed designs [169]. To avoid resource limitations and barriers to trial participation in a rapidly progressive, disabling, and heterogeneous disease, a flexible and scalable multi-arm, the multi-stage trial platform is required.

### The ethical concept of genetic testing must be discussed in view of drug development

We discussed de novo mutations in Section 1–6. In some reports, 21% of patients with ALS, of whom 93% had no family history, carried confirmed pathogenic, or likely pathogenic mutations [120]. Peripheral-blood exome, genome, and Sanger sequencing to identify pathologic mutations in *SOD1* in 4000 patients with ALS from Germany, South Korea, and Sweden has revealed four sporadic ALS cases with de novo mutations in *SOD1*, which might be the therapeutic target of ASO [170]. To avoid missing the opportunity for treatment and earlier confirmed diagnosis, all patients with ALS should be offered genetic counseling and genetic screening. However, the challenges of variant interpretation associated with systematic genetic testing still exist; genetic testing must be accompanied by appropriate genetic counseling with human resources, variant interpretation, limited clinical trial spots, increased request for predictive testing, and psychosocial impact of identifying a genetic variant in patients without family history [129]. The International Consortium for Genetic Testing in ALS Committee was formed in March 2021, aiming to develop global best practice recommendations [129].

## Understanding the molecular pathology for early therapeutic intervention: focus on axonal pathology (Fig. 2)

### What are the pathological processes appropriate for early intervention? Axons are damaged early on

**Fig. 2:**
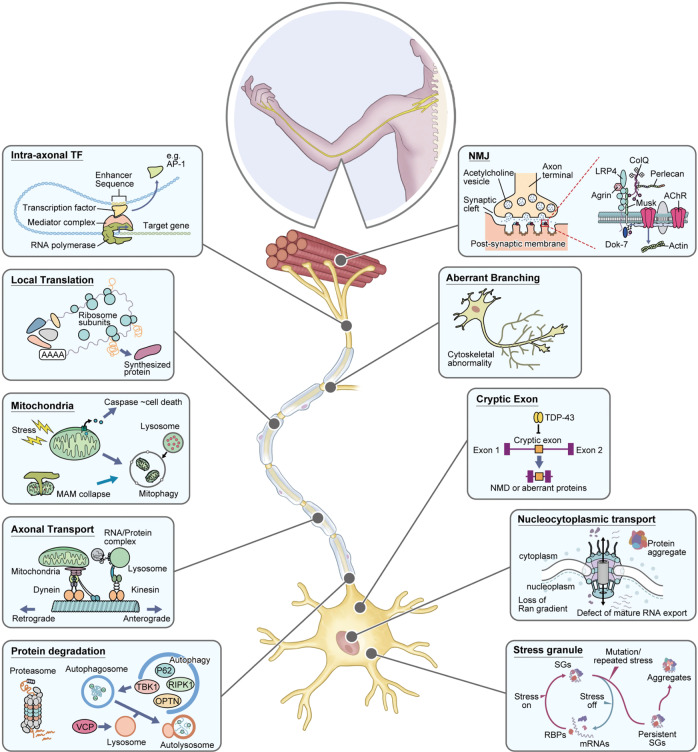
Overview of ALS pathology with a focus on axons. As axons are damaged from the initial stage of ALS, the dying-backward hypothesis, in which motor neurons are damaged from the distal part, has been proposed. RNA-seq of axon fraction shows the presence of intra-axonal transcription factors (e.g., AP-1), although the pathological significance remains unknown. NMJs are the key link between motor neurons and skeletal muscle, and NMJ disconnection is commonly observed in several types of ALS. The local translation is a molecular mechanism necessary for axonal homeostasis. In contrast, when the proteasome and autophagy are dysregulated, abnormal protein aggregation is triggered. Mitophagy is a form of autophagy, and mitochondrial pathology is a common feature of various neurodegenerative diseases. Furthermore, impaired axonal transport impairs the transport of RNA/Protein complex, lysosomes, and mitochondria. Many ALS-causing genes contribute to cytoskeleton function. Morphologically abnormal axon branching has been observed. The pathophysiology of the cell body, which is closely related to the axonal pathology, is also important. Cryptic exon induced nonsense-mediated decay (NMD) or aberrant proteins, persistent stress granules (SGs) formation, and nucleocytoplasmic transport defect have attracted attention as new therapeutic targets

As early/pre-symptomatic diagnosis becomes possible, as described in a previous chapter, intervening in the pathology becomes feasible at earlier stage. Looking back at the failure of the primary efficacy endpoint of a phase III trial of tofersen, interventions in the early stage are also indicated as critical [8]. In ALS animal models, morphological abnormalities of axons and neuromuscular junctions were observed from the early stages of the disease [171, 172]. Spastic paraplegia *(SPG)* type 11, profilin 1, never in mitosis gene A-related kinase 1 *(NEK1)*, tubulin 4a, NFH, and chromosome 21 open reading frame 2 *(C21ORF2* or *CFAP410)* have been identified as ALS-causing and susceptibility genes involved in axonal pathology and cytoskeletal abnormalities by ancestry analysis and GWASs [25, 100, 173]. *C21ORF2* is mutated in ciliopathies [174] and is stabilized by NEK1-mediated hyperphosphorylation and the inability to bind F-box protein 3 [175]. Convergent genetic and experimental data revealed *KANK1* as a new ALS gene and initiation of ALS pathogenesis in the distal axon [98].

Motor neurons are structurally characterized by long axons extending to the tips of the limbs. Focusing on the pathogenesis of both axon compartment and cell bodies may lead to the identification of new therapeutic targets. Mice with *FUS* mutation and hexanucleotide repeat expansion in *C9ORF72* also showed axonal abnormalities, indicating a common pathology in ALS [171]. Abnormalities in axonal morphology and function, as well as the interaction with the extracellular environment to maintain the structure of long axons, are important pathologies that should be clarified as early and specific in motor neurons. In this chapter, we focused mainly on axonal pathology and considered its potential as a novel therapeutic target for ALS.

### Axon sequencing reveals intra-axonal transcription factors

**Fig. 3:**
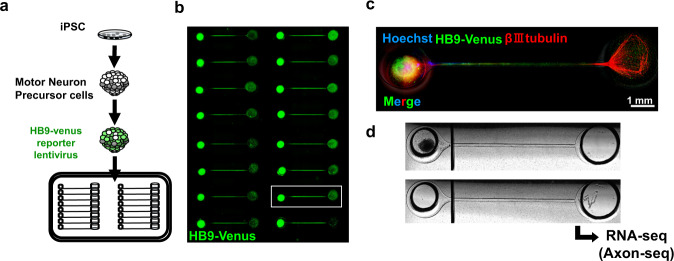
Motor neuron axons were extracted with a microfluidic device. **a** The experimental scheme of MN culture using the microfluidic device. HB9 reporter lentivirus infected motor precursor cells were plated onto the device. **b** Representative ICC images of MNs on the microfluidic device. The axons elongated in the microfluidics to the next well. **c** The enlarged images of nerve organoids stained with βIII-tubulin (cytoplasm) and Hoechst (nuclei). Bar: 1 mm. **d** Representative images of axon dividing. Axons were divided from the SDs by cutting the axon bundle at 450 μm away from the sphere to avoid contaminating the cell body and pushing out due to hydraulic pressure. Modified from ref. [181]

To understand what happens locally in axons, investigating the pathology of axon fraction itself is important. In case of cell culture setting, although several types of microfluidic devices are available on the market, some are specific to cell fraction analysis [176, 177], harvesting a sufficient sample for analysis from the axon compartment remained challenging [178]. A novel microfluidic device with improved dimensions of the well and materials enabled us to perform RNA sequencing using axon fraction (axon-seq) with fewer technical biases with the collection of several macroscopically observable axon bundles. Combining this innovative microfluidic device [179] with patient iPSC-derived motor neuron organoids further revealed the entire profile of the human motor neuron axons (Fig. 3) [180, 181]. This technique identified increased intra-axonal transcription factor, *Fos-B* (AP-1 family member) mRNA as a binding partner of FUS and as a causative event for aberrant axon morphology both in vitro and in vivo [181]. The upregulation of Fos-B mRNA is associated with increased spines [182, 183] and growth cones [184]. Activator protein-1 (AP-1) is increased in a mutant *SOD1*-G93A transgenic mouse model [185] and Fos-B protein accumulate abnormally in the motor neurons of sporadic ALS autopsy samples [181]; thus, dual leucine zipper kinase, the upstream signal protein for c-Jun (another AP-1 family member), and AP-1 family might become a common therapeutic target in ALS [185].

The data obtained may provide important resources for the subcellular fractional analysis of stem cell-derived motor neuron axons. The reproducibility of RNA profiles from the novel microfluidic device [176, 177] justified this approach. Notably, using *TARDBP*-mutated iPSCs, we found another intra-axonal transcription factor, paired-like homeobox protein 2B *(phox2B)*, which showed a lower expression in mutant axons revealed by axon-seq and in situ hybridization [186]. Phox2B knockdown reduced neurite length in human and zebrafish motor neurons [186]. Phox2B-positive ocular motor neurons are resistant to degeneration in ALS compared with spinal motor neurons [187]. Targeted metabolomics identified elevated levels of the arachidonic acid pathway and reduction of arachidonic acid reverse ALS phenotypes in human and Drosophila spinal motor neurons and *SOD1* mouse models [188].

Other groups have revealed the transcriptome of mature myelinated motor axons of the peripheral nervous system using the axon microdissection method devised by Koenig, enabling the isolation of axoplasm RNA to perform RNA-seq analysis [189]. The transcriptome analysis indicated the depletion of glial markers, enriched in neuronal markers and mRNAs related to the cytoskeleton, translation, and oxidative phosphorylation [189].

The whole story of what happens locally in the axon is unclear. Analyzing the molecular pathomechanism of axon fraction, including intra-axonal transcription factor, could provide a clue to elucidate the fragility of motor neurons in ALS.

### Neuromuscular junctions (NMJs) are the key link between motor neurons and the effector skeletal muscle

ALS can be a distal axonopathy disease because many molecular changes of motor neuron degeneration start at NMJs [190]. The NMJ is a highly specialized synapse, which controls the signal between motor neurons and skeletal muscles. Clustering of acetylcholine receptors (AChR) at the NMJ are regulated by signaling molecules such as agrin, low-density lipoprotein receptor-related protein 4 (Lrp4), and muscle-specific receptor tyrosine kinase (MuSK) [191]. In a mutant *SOD1*-G37R transgenic mouse model, NMJ remodeling precedes the loss of the motor unit [192]. The activation of the muscle-specific kinase MuSK by the cytoplasmic protein Dok-7 is essential for NMJ formation, and Dok-7 recovery reduces muscle atrophy in a *SOD1*-G93A transgenic mouse model [193]. The upregulation of mitofusin 2 improves the NMJ morphology of mutant *SOD1*-G93A transgenic mice [194].

TDP-43 accumulation is observed in both intramuscular nerves of sporadic ALS and ALS patient iPSC-derived motor neuron axons [195]. Hyperphosphorylated TDP-43 promotes G3BP1-positive ribonucleoprotein condensate assembly and inhibits local protein synthesis in the axon and NMJ. Dissociation of G3BP1 condensates restores local translation and reduces toxicity by TDP-43 accumulation [195].

FUS mediates the transcriptional regulation of acetylcholine receptors at NMJs and is dysregulated in ALS [196]. Moreover, FUS is involved in NMJ maintenance and axonal transport [197, 198]. The expression of mutant FUS or *FUS* knockdown impairs motor activity and reduces acetylcholine transmission at NMJs in zebrafish [199]. Dipeptide repeat proteins related to C9ORF72 spread between motor neurons and skeletal muscles in vitro and in vivo [200]. Evidence suggests that the pathogenic mechanism of prion protein/exosome transfer is activated in the extracellular space and across the NMJ synapses during the degeneration of the motor cortex with centrifugal spreading [201–203].

Several NMJ microfluidic devices have been developed using human iPSC-derived motor neurons and myotubes [204]. An NMJ chip enables real-time, live imaging of axonal outgrowth, NMJ formation, and muscle maturation, as well as synchronization of motor neuron activity and muscle contraction under optogenetic control for analysis of physiological events [205]. Another important approach is the single-cell transcriptomics of nerve organoids in vitro [206]; pseudo-time analysis or single-cell trajectory analysis can help establish the relationship between the cause and effect of the transcriptome of the organoids [207, 208]. Sophisticated co-culture NMJ organoids can be beneficial for these studies.

### Maintenance of axonal function by local translation

Whether mRNAs found in axon fractions are translated into axons or transported to the nucleus/cell body is an important question. Accumulating evidence has revealed that long motor neuron axons use asymmetrical mRNA localization and rely most strongly on mRNA transport and local translation to maintain homeostasis [209]. The anterior branch of human obturator motor neurons biopsied from patients with ALS demonstrated upregulation of a cluster of genes that play an important role in biological processes involving RNA processing and protein metabolism [210].

The upregulation of ribosome synthesis in axons occurs early in the pathogenesis of both mutant *SOD1*-G93A transgenic mouse models and human ALS autopsy samples, indicating aberrant axonal RNA metabolism [211]. Ribosomal protein mRNAs are locally translated and incorporated into native ribosomes in axons to maintain functional axonal ribosomes, which are reduced in sporadic ALS with TDP-43 pathology [212].

In SMA, reduced SMN decreases the axonal localization of several mRNAs [213] and inhibits the activity of the mammalian target of rapamycin in axons [214]. mTOR translationally alters the cytoskeletal regulator palladin to modulate axon morphogenesis [215]. Moreover, SMN regulates axonal localization and local translation of *growth-associated protein 43* mRNA in growth cones through HuD and insulin-like growth factor 2 [216]. The aberrant distribution of SMN in cytosolic FUS accumulations reduces SMN in axons [217, 218]. The accumulation of mutant human FUS induces an integrated stress response and reduces protein synthesis in nearby axons [219]. Moreover, *FUS* mutation affects nonnuclear pools of splicing factor proline and glutamine-rich [220], which has been found to orchestrate spatial gene expression and is essential for axonal viability [221, 222].

Another example is casein kinase 2 alpha (CK2a). CK2a phosphorylates and triggers G3BP1 stress granule-like structure disassembly in injured axons [223]. CK2a activity is temporally and spatially regulated by the local translation of mRNA in axons after injury [223]. Axoplasmic calcium concentration is a determinant of the translational activation of different axonal mRNA and regenerative axonal growth [224].

Thus, local translation plays an important role in axonal homeostasis and nerve regeneration, which are dysfunctional in ALS.

### Protein degradation: regulation of abnormal protein aggregation by proteasome and autophagy

The homeostatic processes engaged in eliminating defective organelles and aggregated proteins include autophagy and ubiquitin-proteasome systems. The accumulation of SOD1 and TDP-43 has been observed in autopsy specimens. Disruption of protein homeostasis has long been considered a pathogenic mechanism in ALS.

In the experimental models, constitutive autophagy in neurons maintains cellular homeostasis by balancing protein synthesis and degradation, particularly within the distal axonal processes [225, 226]. Disruption of the endosomal-lysosomal system by loss of Alsin deteriorates the phenotype of *SOD1*-H46R transgenic mice by accelerating the accumulation of misfolded proteins and immature vesicles in the spinal cord [19]. *FUS* mutation causes axonal retention of the FUS protein before its aggregation triggered by poly(ADP-ribose) polymerase-dependent DNA dependent repair signaling [227].

Optineurin is involved in autophagy and protein degradation pathways [17]. Optineurin binds to ubiquitin and regulates necrosis factor-kappa B activation and apoptosis [228]. Receptor-interacting kinase 1 (RIPK1)-dependent signaling is suppressed by optineurin by regulating its turnover [18]. Optineurin loss leads to progressive demyelination and axonal degeneration by activating necroptotic machinery in the central nervous system [18].

Moreover, optineurin is involved in several selective autophagy processes regulated by TBK1 [229]. *TBK1* mutations are associated with impaired binding of autophagy adapter proteins. TBK1 phosphorylates and activates the Smith–Magenis syndrome chromosome region, candidate 8 (SMCR8), a member of the C9ORF72 complex, activating the autophagy pathway via RabGTPase [230]. FUS protein accumulation in autopsy cases with optineurin mutations [231] and decreased expression of TBK1 [232] suggest crosstalk between the disruption of protein homeostasis and abnormal RNA metabolism.

In GWAS, a meta-analysis of multiracial sporadic ALS data identified the *GPX3-TNIP1* region, which encodes antioxidant glutathione peroxidase 3, and tumor necrosis factor-induced protein 3 interacting protein 1, a protein that interacts with optineurin [233]. Furthermore, mutations in the LC domain region of *T cell-restricted intracellular antigen 1* suppress stress granule degradation and abnormal accumulation of TDP-43, which may be a cause of ALS [234]. Cyclin-F is a cell cycle regulator and ubiquitin E3 ligase [235] and interacts with SQSTM1/p62 in the autophagy pathway [236]. In zebrafish, a variant in *UBQLN4* compromises motor axon morphogenesis, impairing proteasomal function [237, 238].

The identification of these genes indicates that the disruption of protein homeostasis is an important common mechanism in ALS, especially in the compartment of motor neuron axons.

### Mitochondrial pathology

The mitochondria generate adenosine triphosphate through oxidative phosphorylation and provide the axonal energy demand [239]. After synthesis at the cell body, the mitochondria accumulate at the nodes of Ranvier to meet metabolic needs [240]. Several neurodegenerative diseases are affected by disrupted mitochondrial activity, transport proteins, and microtubule association [241]. Mutations in *RAPGEF2* impair microtubule stability and mitochondrial distribution in axons [242]. Dysfunction in Rho GTPase 1 (Miro 1), the outer mitochondrial membrane protein, leads to anterograde axonal transport defects [243]. The imbalance between mitochondrial fission and fusion leads to abnormal morphology of the mitochondria in spastic paraparesis [244]. Syntaphilin, a mitochondria-anchoring protein, mediates the clearance of dysfunctional mitochondria from motor neuron axons [245]. Loss-of-function mutations in *SIGMAR1* decrease mitochondria-associated membrane (MAM), impairing retrograde transport and axonal degeneration [246, 247].

Phosphatase and tensin homologs deleted from chromosome 10-induced kinase 1 (PINK1) and parkin are key regulators of mitophagy, a selective autophagic pathway to eliminate dysfunctional mitochondria [248]. The disruption of PINK1 signaling is found in *SOD1* mutant mice and samples from patients with sporadic ALS [249]. *Parkin* expression is regulated by TDP-43 and reduced in motor neurons of TDP-43 pathology with ALS [250, 251]. Axonal transport of mitochondria is disrupted in *FUS*-mutant patients with aggregation of Parkin and PINK1 [252].

RNA-seq revealed reduced gene expression of mitochondrially encoded electron transport chain transcripts, and neuropathological analysis of *C9ORF72*-mutated ALS postmortem tissue confirmed selective dysregulation of mitochondrially encoded transcripts in ventral horn spinal motor neurons [253]. Genetic manipulation of mitochondrial biogenesis in C9ORF72 motor neurons corrected the bioenergetic deficit and rescued the axonal length and transport phenotypes [253].

The degradation of autophagic vacuoles that engulf damaged mitochondria is impaired in distal axons in a *SOD1*-G93A transgenic mouse model [254]. A potential drug that reduced neuronal cell death in a *SOD1*-G93A mouse model is a neuronal SIGMA1-receptor agonist (SA4503), which reduces oxidative stress and regulates calcium flux in the mitochondria [255]. Pridopidine, a SIGMA1 agonist, is being investigated in the HEALEY ALS Platform Trial (NCT04615923). Clinical trials of the combination of dextromethorphan and quinidine, which affect the demethylation of the P450 cytochrome enzyme, have revealed improvement in the pseudobulbar effect in patients with ALS [256]. Mitochondrial activation drugs, such as MA-5 [257], have potential as a strategy for various mitochondrial dysfunction pathologies.

Mitochondrial metabolism is the emerging and noteworthy therapeutic target in ALS.

### Axonal transport

Intracellular transport of cargo is especially important in neurons because of the polarization between axon and cell bodies [258]. RNA/protein complexes and organelles, such as the mitochondria, are synthesized in the soma and transported along the axon. The distribution of this cargo at the right time and place in the axon depends on the proper transportation of the cargo. The transport defect was revealed in ALS, and the proximal axons of large motor neurons harbor abnormal accumulation of mitochondria, phosphorylated neurofilaments, and lysosomes [259–262]. Furthermore, the structure of spheroids in motor nerve axons in ALS autopsy samples contains different types of vesicles, neurofilaments, lysosomes, mitochondria, and microtubules [258, 263], suggesting axonal transport reduction.

Aberrant cargo transport within axons occurs early in ALS disease progression in mutant *SOD1*-G93A transgenic mouse models of ALS [264–266]. Inhibiting p38 MAPK rescues retrograde cargo transport defects within axons of mutant *SOD1*-G93A transgenic mouse models [267]. Moreover, ALS-related mutations in TDP-43 alter the transport function [268]. Similarly, defects in cargo transport within axons were demonstrated in *FUS*-mutant iPSC-derived motor neurons, which were rescued by inhibiting histone deacetylase 6 [269].

Dynactin 1, which binds to microtubules, is a motor protein responsible for the retrograde transport of various proteins and vesicles [270]. ALS and slowly progressing, autosomal dominant, distal hereditary motor neuropathy in vocal paresis are due to loss-of-function mutations in dynactin 1 [271–273]. Kinesin is another motor protein involved in the anterograde axonal transport [274]. Its family member 5A (KIF5A) is mutated in the N-terminal motor domain in SPG10 and CMT type 2, whereas the C-terminal domain is mutated in ALS [275]. Patients with loss-of-function *KIF5A* mutations have longer survival times than those with typical ALS [276, 277]. Furthermore, loss-of-function mutations in *KIF1A* are present in the motor or neck domains [278, 279]. These motor proteins are dysregulated in sporadic ALS pathology [280]. Loss-of-function mutation in *KIF5A* is found in 0.12% of sporadic ALS in Japan [281].

Annexin A11, a phosphoinositide-binding protein associated with RNA granules, functions as a molecular tether between lysosomes and RNA granules. Such tethering is impaired by ALS-associated *annexin A11* mutations [282, 283]. Late endosome-bearing mRNAs encoding mitochondrial functional molecules stop at the mitochondria, and these mRNAs are translated on Rab7a endosomes locally in the axon [284]. Axonal transport and other pathological processes, such as autophagy and mitophagy, closely interact. Defects in the cargo transport within axons are common in various neurodegenerative diseases [285].

### Aberrant cytoskeleton and axon branching

Several variants of the gene encoding *α-tubulin* destabilize the microtubule network and reduce the repolymerization capability of the cytoskeleton [173]. Mutations in *profilin 1*, which converts monomeric actin to filamentous actin, lead to familial ALS. Ubiquitinated aggregates, including TDP-43, are present in cells that express mutant profilin 1 [286]. Reduced binding with actin and axon growth are observed in mutant *profilin 1*-expressing cells. *Profilin 1* transgenic mice have been observed to recapitulate the phenotype of MNDs [287]. C9ORF72 modulates the activity of small GTPases, increases the activity of LIM kinases 1 and 2, and regulates axonal actin dynamics [288]. Various actin isoforms are expressed in primary mouse motor neurons, and their transcripts are translocated into motor neuron axons [289]. NFL transcripts are reduced in ALS [290]. Moreover, neurofilaments are found in a spheroid structure (large axonal swelling) [291]. NFL and pNFH are also known as biomarkers for ALS [158, 159]. Thus, cytoskeleton abnormal morphologies contribute to the pathogenesis of ALS. Non-labeled live imaging of stimulated Raman scattering microscopy [292] can visualize peripheral degeneration in live ALS mouse models and human postmortem tissue [171]. The novel technology might be a supportive tool for diagnosing cytoskeletal abnormalities much earlier and assessing the effectiveness of the therapies.

TDP-43 is a crucial splicing repressor, and its loss results in novel cryptic exons being erroneously included in mature mRNA. One of the splicing targets of TDP-43, STMN2, a regulator of microtubule stability, is involved in the pathomechanism of *TARDBP* mutations [137, 293]. STMN2 is decreased following *TARDBP* knockdown due to altered splicing when TDP-43 is mislocalized and in motor neurons from patients and the spinal cord of postmortem samples. Posttranslational STMN2 stabilization rescues neurite outgrowth and axon regeneration deficits by depleting TDP-43 [137]. QurAlis QRL-201 is a therapeutic ASO that restores STMN2 mis-splicing due to TDP-43 pathology.

Axonal branching is a key mechanism of synaptic plasticity [294]. Aberrant axonal branching is implicated in the pathomechanisms of ALS. For example, motor neurons cultured from mutant *SOD1*-G93A transgenic mouse models exhibit enhanced axonal branching [295]. The overexpression of mutant human *TARDBP* in zebrafish embryos induces a phenotype that includes shorter motor neuron axons, premature and increased axonal branching, and ends in abnormal swimming [296]. Progranulin rescues mutant *TARDBP*-induced aberrant axonal branching and short axonal outgrowth [297]. Branching in *FUS*-mutant motor neuron axons is increased compared with that in isogenic controls in vitro [181]. This phenotype was confirmed using other ALS causative mutations, including *SOD1* and *TARDBP* [181]. Morphological changes in motor neuron axon branching have been found to precede motor neuron death in mutant *SOD1*-G93A transgenic mouse models [171], and abnormal neural branching has been detected in zebrafish that overexpress mutant *FUS* [199]. Moreover, other groups have reported increased axon branching in *FUS, SOD1*, and *TARDBP* mutant iPSC models [298]. *SMN* knockdown in zebrafish embryos significantly increases motor neuron branching [299]. Furthermore, mutant *CCNF* zebrafish developed a motor neuron axonopathy, which consists of shortened primary motor neuron axons and an increased frequency of aberrant axonal branching [235, 300].

The meaning of axonal branching might be different in each developmental stage [301]. In the embryonic stage, axon pathfinding and synaptic formation are important. However, in the developed stage, aberrant axon branching might have a disadvantage in terms of the normal function of electronic neurotransmission. The significance of aberrant axonal branching in the context of the neurodegenerative model in vivo must be elucidated.

### The pathology of the cell body is also important: persistent stress granule formation and nucleocytoplasmic transport

Although we have focused on axonal pathology, considering the pathology occurring in motor neuron cell bodies is also important to understand the overall picture of ALS. FUS and TDP-43, which normally reside in the nucleus, have RNA-binding domains that regulate RNA metabolism and transport [302]. Mutant TDP-43 and FUS change their localization from the nucleus to the cytoplasm [303], suggesting that they gain or lose function in the nucleus [304] and are important in normal physiological aging [304]. Stress granules are droplets that form under various stresses, such as heat shock and hypoxia, and are composed of mRNA and RNA-binding proteins [305]. They protect cellular homeostasis by temporarily inactivating mRNA translation under stress and allowing it to resume after the stress is removed. TDP-43, FUS, hnRNPA1, and hnRNPA2 are nuclear RNA-binding proteins with prion-like domains and undergo LLPS to form functional liquids, including stress granules, which can be converted into abnormal hydrogels that contain pathological fibrils often seen in neurodegenerative diseases [306–309]. Mutations in prion-like domains increase the rate of fibril formation and initiate disease [310]. Karyopherin-β2 (also known as transportin-1) binds the proline-tyrosine nuclear localizing signal and then blocks and reverses FUS fibril formation [310–313]. Moreover, importin-α and karyopherin-β1 block and reverse TDP-43 fibril formation [310]. The identification of molecules involved in the assembly/disassembly of LLPS is being actively pursued because the identification of molecules that regulate the dissociation of LLPS droplets in cells may help avoid aggregation toxicity [314–316].

Alterations in nucleoporins and the function of nuclear pore complexes have also been considered a therapeutic target in ALS [317–319]. RanGAP1 regulates the directionality of the transport [320]. If RAN gradients are impaired, cytoplasmic accumulation of nuclear proteins, as well as defected export of mature RNAs from the nucleus, sequesters nucleocytoplasmic transport [319]. Endosomal sorting complexes required for transport machinery, including charged multivesicular body protein 7 (CHMP7), is involved in proteasomal degradation of disassembled nucleoporins [321]. Inhibiting the nuclear export of CHMP7 triggers nucleoporin reduction, and TDP-43 dysfunction and knockdown of CHMP7 alleviate Ran GTPase mis-localization [322]. Moreover, mutated FUS interacts with nucleoporins and declines nucleocytoplasmic transport in Drosophila and iPSCs [323]. Toxic proline:arginine dipeptides from C9ORF72 bind to karyopherin-β2 and impede nucleocytoplasmic transport by interacting with nuclear import receptors [324].

Dying-forward and dying-backward hypotheses are both important [325, 326]. The association between axonal dysfunction and these cell body/nuclei events, including nucleocytoplasmic transport and stress granule/aggregation formation, should be elucidated in the context of axonal pathology.

## Conclusion

With the development of edaravone as the second ALS drug after riluzole, the treatment of ALS has made steady progress based on the knowledge gained from the elucidation of the pathogenesis of familial ALS. Comprehensive analyses of causative genes, disease susceptibility genes, and disease-modifying genes will continue to be important for the complete understanding of the disease. Targeting axons, the early pathological site of ALS, is also desirable. In the future, there is a strong need for developing more effective therapies to prevent neurodegeneration and symptomatic treatments to alleviate the disease. Like nusinersen for SMA, expectations for ASO remain high.

With the paradigm shift in therapeutic development, we must debate ethical issues, such as genetic diagnosis, in sporadic ALS. Because of the impact on the patient and the family, genetic diagnosis also places a heavy psychological burden on the attending physician. Even if a treatment is developed in the future, prenatal diagnosis will make it even more difficult to make ethical choices. Even if the guidelines can provide examples, each particular case will be subject to difficult decisions. It will also be necessary to take into accounts the mental exhaustion of not only the patient and family but also the medical profession.
